# Endothelial Nitric Oxide Synthase and Superoxide Mediate Hemodynamic Initiation of Intracranial Aneurysms

**DOI:** 10.1371/journal.pone.0101721

**Published:** 2014-07-03

**Authors:** Nicholas Liaw, Jennifer M. Dolan Fox, Adnan H. Siddiqui, Hui Meng, John Kolega

**Affiliations:** 1 Toshiba Stroke and Vascular Research Center and Department of Mechanical and Aerospace Engineering, State University of New York, Buffalo, New York, United States of America; 2 Toshiba Stroke and Vascular Research Center and Department of Neurosurgery, State University of New York, Buffalo, New York, United States of America; 3 Toshiba Stroke and Vascular Research Center and Departments Neurosurgery and Radiology, State University of New York, Buffalo, New York, United States of America; 4 Toshiba Stroke and Vascular Research Center and Departments of Mechanical and Aerospace Engineering, Neurosurgery, and Biomedical Engineering, State University of New York, Buffalo, New York, United States of America; 5 Toshiba Stroke and Vascular Research Center and Department Pathology and Anatomical Sciences, State University of New York, Buffalo, New York, United States of America; University of Iowa, United States of America

## Abstract

**Background:**

Hemodynamic insults at arterial bifurcations are believed to play a critical role in initiating intracranial aneurysms. Recent studies in a rabbit model indicate that aneurysmal damage initiates under specific wall shear stress conditions when smooth muscle cells (SMCs) become pro-inflammatory and produce matrix metalloproteinases (MMPs). The mechanisms leading to SMC activation and MMP production during hemodynamic aneurysm initiation are unknown. The goal is to determine if nitric oxide and/or superoxide induce SMC changes, MMP production and aneurysmal remodeling following hemodynamic insult.

**Methods:**

Bilateral common carotid artery ligation was performed on rabbits (n = 19, plus 5 sham operations) to induce aneurysmal damage at the basilar terminus. Ligated animals were treated with the nitric oxide synthase (NOS) inhibitor LNAME (n = 7) or the superoxide scavenger TEMPOL (n = 5) and compared to untreated animals (n = 7). Aneurysm development was assessed histologically 5 days after ligation. Changes in NOS isoforms, peroxynitrite, reactive oxygen species (ROS), MMP-2, MMP-9, and smooth muscle α-actin were analyzed by immunohistochemistry.

**Results:**

LNAME attenuated ligation-induced IEL loss, media thinning and bulge formation. In untreated animals, immunofluorescence showed increased endothelial NOS (eNOS) after ligation, but no change in inducible or neuronal NOS. Furthermore, during aneurysm initiation ROS increased in the media, but not the intima, and there was no change in peroxynitrite. In LNAME-treated animals, ROS production did not change. Together, this suggests that eNOS is important for aneurysm initiation but not by producing superoxide. TEMPOL treatment reduced aneurysm development, indicating that the increased medial superoxide is also necessary for aneurysm initiation. LNAME and TEMPOL treatment in ligated animals restored α-actin and decreased MMPs, suggesting that eNOS and superoxide both lead to SMC de-differentiation and MMP production.

**Conclusion:**

Aneurysm-inducing hemodynamics lead to increased eNOS and superoxide, which both affect SMC phenotype, increasing MMP production and aneurysmal damage.

## Introduction

The initiation of intracranial aneurysms (IAs) is multifaceted, and hemodynamic insult is believed to be a causative factor [1]–[4]. The preferential location of IAs to the apices of bifurcations or outer curvatures of blood vessels coupled with a number of clinical observations of de novo aneurysm formation following carotid occlusion, with incidences ranging form 0.7 to 20% [1], [5]–[7], have implicated increased blood flow in the genesis of IAs. To study hemodynamic mechanisms of IAs, we have developed a rabbit basilar terminus (BT) IA model by ligating both common carotid arteries to increase flow through the basilar artery [8], [9]. A striking response at the BT is extensive loss of internal elastic lamina (IEL), which occurs as early as 2 and 5 days post flow increase [9], [10] and localizes to regions of the apex experiencing high wall shear stress and flow acceleration [11]. The IEL loss and wall thinning is attributed to matrix metalloproteinases (MMPs), that include MMP-2 and MMP-9 [10]. Interestingly, smooth muscle cells (SMCs), not infiltrating macrophages, are the source of MMPs in the vascular wall early-on in flow-induced aneurysm initiation [12]. The mechanisms leading to SMC activation and their MMP production are unknown.

Previous studies have highlighted critical, yet contradictory roles for nitric oxide (NO) in regulating MMP induction in flow-dependent remodeling [13], [14]. Inhibiting NO synthesis decreased MMPs and reduced adaptive outward expansion in chronic flow-loaded arteries [14]. However, knockout of eNOS in hypertensive rodents resulted in increased MMPs and IA formation [13]. On the other hand, superoxide and other reactive oxygen species (ROS) (e.g., peroxynitrite, a reaction product of NO and superoxide) induce MMP production [15] and activation [16] and also trigger SMC phenotype changes [17], [18]. In rodents with unilateral carotid ligation, hypertension, and matrix weakening, scavenging for ROS reduced aneurysm formation. Thus NO or superoxide and/or their reaction product, peroxynitrite, are potential effectors of MMPs during aneurysm initiation triggered by hemodynamics.

Given the widely acknowledged roles of NO and superoxide in flow-mediated remodeling, we sought to determine if NO and/or superoxide are important in hemodynamic-driven IA initiation. Specifically, we asked if changes in these molecules lead to SMC de-differentiation, marked by loss of α-actin and MMP-2 and -9 production, and subsequent IA initiation. To this end, we inhibited NO production and scavenged superoxide to test the role of these respective molecules during aneurysm initiation. This study sheds light on the signals that induce production of MMPs in IAs, which could provide additional avenues by which to manipulate disease progression.

## Methods

### Ethics Statement

All procedures were approved by the Institutional Animal Care and Use Committee of the State University of New York at Buffalo (protocol #NSG22112Y “Hemodynamic Induction of Pathologic Remodeling Leading to Intracranial Aneurysms”, Hui Meng, principal investigator).

### Hemodynamic Induction of IA

IAs were induced at the BT in sexually mature (6–8 month old), unmated, female New Zealand White rabbits (n = 19) via bilateral ligation of the common carotid arteiress as previously described [8], [9], [11]. In rabbits, the cerebral circulation is fed through three major vessels: the common carotid arteries and the basilar artery. When both carotid arteries are ligated, blood is re-routed through the basilar artery to restore perfusion throughout the brain thus causing a compensatory flow increase in the basilar artery [8], [9], [11], [19]. We previously determined that bilateral carotid ligation consistently results in a 400 to 900% flow increase through the basilar artery, as measured with transcranial Doppler ultrasonograohy [8], [19]. Flow increase through the basilar artery results in loss of the IEL at the BT visible within 2 days and progressive media thinning and bulge formation [8], [9], with thin-walled, wide-necked bulges being seen 6 months after ligation [9]. These bulges localize to a similar location and with similar histopathological characteristics as human aneurysms. In sham-operated animals (n = 5), the carotids were exposed, but not ligated.

### NOS Inhibition and Superoxide Scavenging

**Figure 1:**
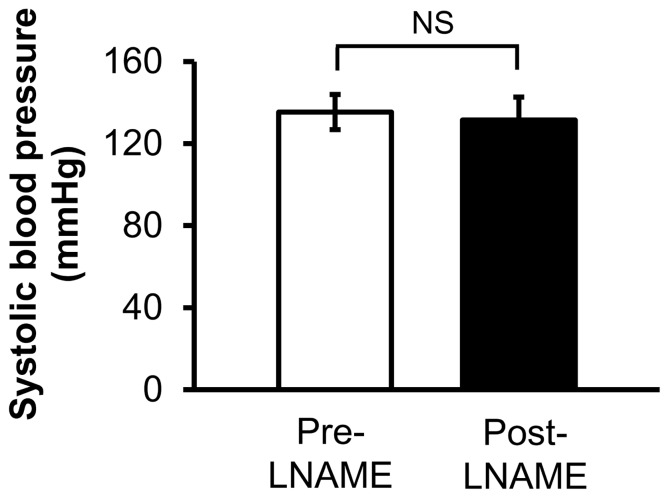
Systolic blood pressure of ligated animals treated with LNAME. The average blood pressure in rabbits was taken pre- and post- LNAME administration. LNAME treatment does not change blood pressure. NS indicates no statistically significant difference (p = 0.68, Mann-Whitney U-test).

The L-arginine derivative, N(G)-nitro-L-arginine-methyl ester (LNAME), was used to inhibit NOS activity in one group of bilaterally carotid-ligated rabbits (n = 7). Animals were given subcutaneous injections of LNAME dissolved in normal saline at a dose of 100 mg/kg/day. Because high doses of LNAME can induce high blood pressure (Guzman), which in turn, can induce vascular remodeling [20], blood pressure was measured in animals treated with LNAME using a forelimb cuff sphygmomanometer before drug treatment and before sacrifice, to confirm that drug treatment did not induce high blood pressure (Figure 1, p = 0.68). In normotensive animals, LNAME can inhibit NOS activity [21] and flow-induced vascular remodeling [21], [22].

In a separate group (n = 5), ligated rabbits were given intraperitoneal injections of the superoxide scavenger 1-oxyl-2,2,6,6-tetramethyl-4-hydroxypiperidine (TEMPOL). TEMPOL was dissolved in normal saline and administered at 250 mg/kg/day. For both drugs, injections began one day before ligation surgery and were subsequently administered daily until sacrifice 5 days later. Both LNAME and TEMPOL groups were compared to ligated rabbits receiving no drug treatment (n = 7).

### Histology and Aneurysm Scoring

All animals were sacrificed 5 days after ligation by intravenous injection of sodium pentobarbital (100 mg/kg). Upon sacrifice, the vertebral arteries were perfused with 1 U/mL heparinized saline for 10 minutes and pressure fixed at 150 mmHg with 10% phosphate buffered formalin for 10 minutes. Brains were extracted from the animals and placed in 10% phosphate buffered formalin for at least 24 hours. The BT was excised, embedded in paraffin, and sectioned longitudinally at a 4 µm thickness. Slides were stained with Van Gieson stain and the slide with the most damage from each animal was used to assess degree of IA damage. Adjacent slides were used to assess molecular changes via immunofluorescence.

To access the degree of aneurysm damage, 3 histological characteristics of IAs (IEL loss, media thinning, and bulge formation) were measured in Van Gieson stained slides of the BT as previously described [9], [12]. Briefly, all three characteristic were measured as the sum of lengths >50 µm along the vessel wall that met the following criteria: IEL loss was defined as complete absence of IEL; media thinning was defined as >30% loss of media thickness (compared to the average media thickness measured downstream from the BT); and bulge formation was defined as gaps where the vessel wall deviated from tubular vascular structure. Three blinded observers performed histological examinations independently, and their individual data were averaged to generate the final measure for each observation. An aneurysm development score (ADS) was used to quantify the degree of IA damage in the slides, as described previously [9]. The ADS is defined as the sum of the lengths of vessel wall where all three IA characteristics are present simultaneously, multiplied by the percentage of media thinning and divided by the BA diameter. An increase in the score indicates progressive aneurysmal development.

### Immunohistochemistry and Quantitation

For immunofluorescence analysis, sections were de-paraffinized, rehydrated, and boiled in 10 mM citrate buffer (pH 6.0) for antigen retrieval. Sections were blocked in 5% normal donkey serum, and subsequently stained with primary antibodies against the following: eNOS (R&D, AF950), inducible NOS (iNOS; BD Transduction Labs, 610328), nNOS (Abcam, AB72428), nitrotyrosine (a marker for peroxynitrite; Chemicon, MAB5404), 8-hydroxyguanosine (a marker for ROS; Abcam, AB62623), MMP-2 (Millipore, MAB3308), or MMP-9 (Millipore, MAB3309). Samples were then incubated with donkey secondary antibodies conjugated with Alexafluor 647 (Jackson Immunoresearch), mounted with 4,6-diamidino-2-phenylindole (DAPI) containing media (Vector Labs) and imaged with a Zeiss Axio Imager Z1 microscope at 20x magnification. Sections were also co-stained for smooth muscle α-actin (Abcam) and β-actin (Abcam) and visualized with donkey anti-mouse rhodamine-conjugated and bovine anti-goat DyLight Fluor 649-conjugated secondaries (Jackson Immunoresearch), respectively.

To quantify immunofluorescence, average intensity was measured in defined regions of interest using ImageJ software (National Institutes of Health). To minimize inter-slide variability, specimens for any given molecular staining were stained at the same time. These slides were also imaged in a single session under identical conditions of magnification, illumination and exposure time. The background signal was subtracted from each image using Image-J rolling ball method with a defined radius of 50 pixels. Further noise reduction was achieved by excluding pixels as indistinguishable from background if they were less than one standard deviation from the mean pixel intensity of the entire image. The region of interest for analysis was defined as a 800 µm length of vessel wall centered at the BT. This length has been empirically determined to include the zone of IA damage in the majority of slides. Staining intensity was then measured in the media and intima by manually outlining these regions to segment them from the auto-fluorescent IEL and surrounding tissue. MMP-2 and MMP-9 staining were quantified as the percent of tissue that stained positive, which was determined by taking the area of the region of interest, in which staining intensity exceeded the mean image intensity of the whole image plus one standard deviation, and dividing by the area of the region of interest.

### Statistical analysis

All values are expressed as mean ± standard error of mean. Statistical analyses were performed using Minitab 16 software (Minitab Inc.). Mann-Whitney U tests were used to test for statistical significance for all comparisons. For all statistical tests, differences were considered significant at p≤0.05. In circumstances where statistically significant differences were found in immunofluoroescent staining or in the overall ADS, a post hoc power analysis was performed using SigmaXL. Table 1 provides a summary of statistical power.

**Table 1:** Sensitivity as determined using post hoc power analysis.

Assessment	Comparison	Figure	Power (1-Beta)
ADS	Ligated vs. Ligated+LNAME	2	0.95
eNOS	Sham vs. Ligated	3	0.99
8-Hydroxyguanosine	Sham vs. Ligated	5	0.99
ADS	Ligated vs. Ligated+TEMPOL	6	0.83
8-Hydroxyguanosine	Ligated vs. Ligated+Tempol	6	0.86
Alpha Actin	Sham vs. Ligated	7	0.99
Alpha Actin	Ligated vs. Ligated+LNAME	7	0.86
Alpha Actin	Ligated vs. Ligated+TEMPOL	7	0.86
MMP2	Sham vs. Ligated	7	0.84
MMP2	Ligated vs. Ligated+LNAME	7	0.95
MMP2	Ligated vs. Ligated+TEMPOL	7	0.82
MMP9	Sham vs. Ligated	7	0.73
MMP9	Ligated vs. Ligated+LNAME	7	0.70

## Results

### NOS inhibition decreased IA initiation

**Figure 2:**
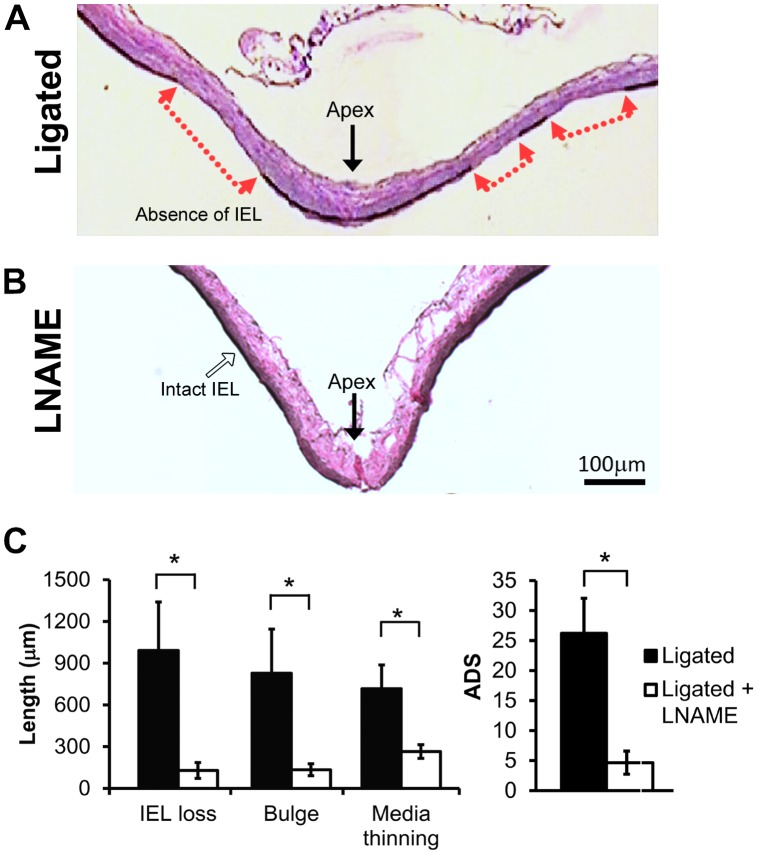
LNAME inhibited flow-induced aneurysm initiation at the BT. (A) Van Gieson’s stain of the basilar terminus (BT) 5 days after bilateral common carotid artery ligation shows a long region of the vessel wall (between red arrows) exhibiting IEL loss, bulge formation and medial thinning. (B) BT in LNAME-treated animals 5 days after ligation had intact IEL and little bulge formation and medial thinning. Scale bars = 100 µm. (C) The lengths of IEL loss, bulging, media thinning, and the composite aneurysm development score (ADS) were all reduced with LNAME treatment as compared to untreated ligated animals. Bars represent mean ± standard error. *Statistically significant difference between ligated and LNAME-treated groups (p<0.05, Mann-Whitney U-test).

All rabbits with bilateral carotid ligation (n = 7) experienced increased flow as described [8], [9], [11], [19] and verified by transcranial Doppler ultrasonography measurements. Ligated animals exhibited aneurysmal damage at the BT 5 days after surgery, as we have previously reported [9]. Changes included IEL loss, media thinning, bulge formation, and increased ADS (Figure 2A). To determine if NOS activity contributed to the aneurysmal damage, an additional 7 carotid ligated rabbits were treated with LNAME, an inhibitor of NOS activity. Van Gieson’s stain of the BT showed decreased IA damage in LNAME-treated animals compared to non-drug treated rabbits. Specifically, IEL was nearly intact in LNAME-treated animals (Figure 2B), whereas untreated ligated animals had large zones of IEL loss (Figure 2A). Furthermore, individual markers of aneurysm initiation – IEL loss, aneurysm bulge length and media thinning – were reduced by LNAME (Figure 2C; p = 0.007, p = 0.005 and p = 0.007, respectively), and the composite ADS was significantly lower with LNAME treatment (p = 0.005).

### IA hemodynamics increased eNOS, but not nNOS or iNOS

**Figure 3:**
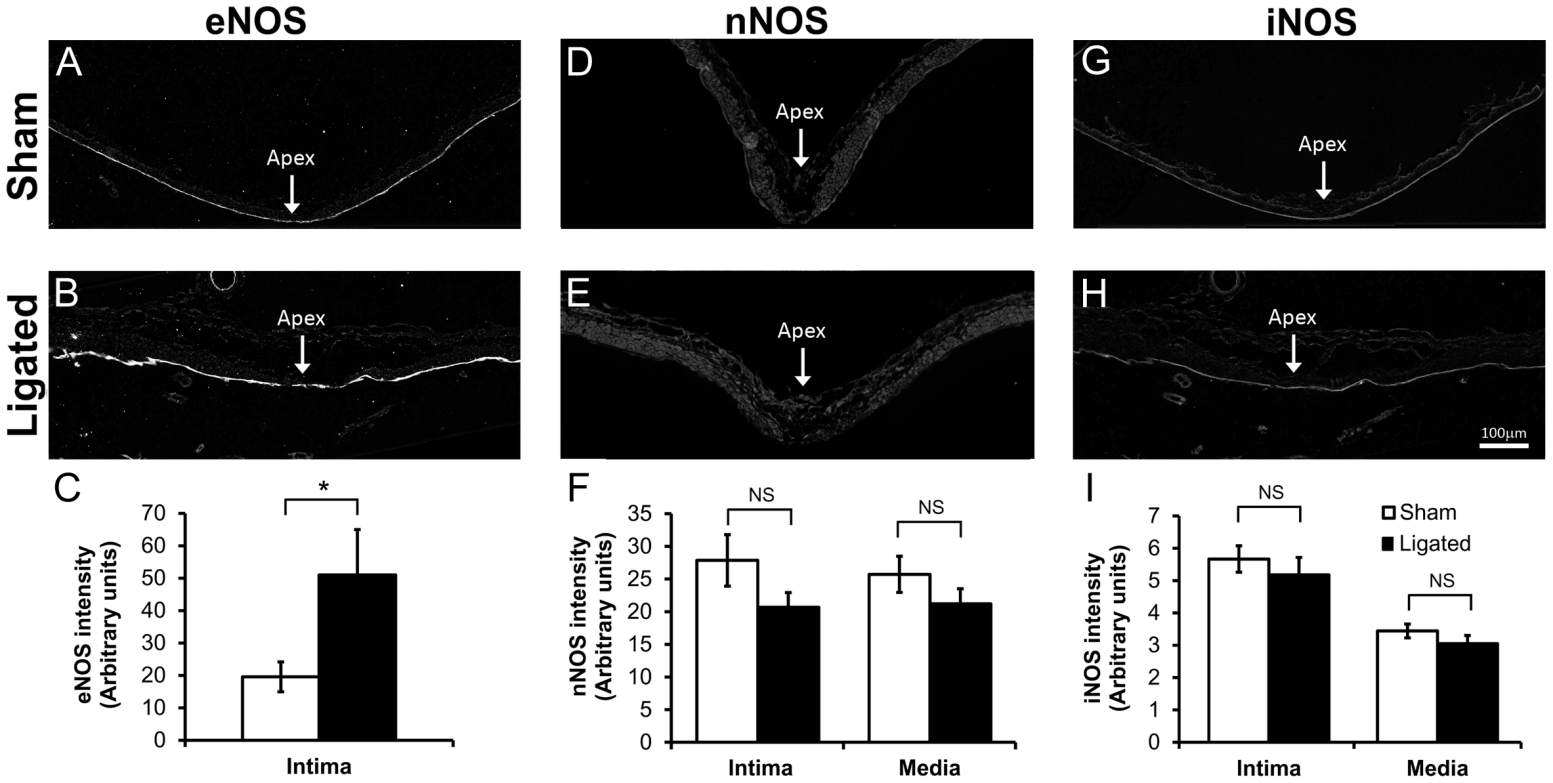
eNOS increased in the intima of the BT 5 days after ligation. eNOS (A, B), nNOS (D, E) and iNOS (G, H) proteins were detected by indirect immunofluorescence at the BT in sham-operated (A, D, G) and carotid-ligated animals (B, E, H). Scale bar = 100 µm. eNOS was significantly higher in the intima of 5-day ligated animals compared to sham-operated animals (C). In contrast, nNOS (F) and iNOS (I) showed slightly lower staining in both the intima and media of 5-day ligated animals; however these differences did not reach statistical significance. Bars represent mean ± standard error. *Statistically significant difference between ligated and sham surgery groups (p<0.05, Mann-Whitney U-test). NS indicates no significant difference.

To determine potential sources of NOS activity that were inhibited by the LNAME treatment, the BT was stained for eNOS, nNOS, and iNOS protein (Figure 3). Immunofluorescent staining for eNOS was localized to the intima as expected (Figure 3A and 3B). The intensity of eNOS staining in the intima was significantly higher in 5-day ligated animals compared to sham-operated animals (Figure 3C; p = 0.037). In contrast, nNOS was weakly expressed in the artery wall and was more prevalent in the media. There was little difference in nNOS staining of the BT in between ligated and sham-operated animals (Figure 3E and 3D). A small decrease in nNOS staining was measured in both the intima and media of the 5-day ligated rabbits, but this change was not significant (Figure 3F; p = 0.21 and p = 0.21, respectively). iNOS staining was unremarkable in both sham-operated and ligated animals (Figure 3G and 3H) and no difference in staining was detected in the intima or the media (Figure 3I; p = 0.54 and p = 0.27, respectively). Thus eNOS may be the important isoform in aneurysm initiation.

### IA-initiating hemodynamics did not affect peroxynitrite

**Figure 4:**
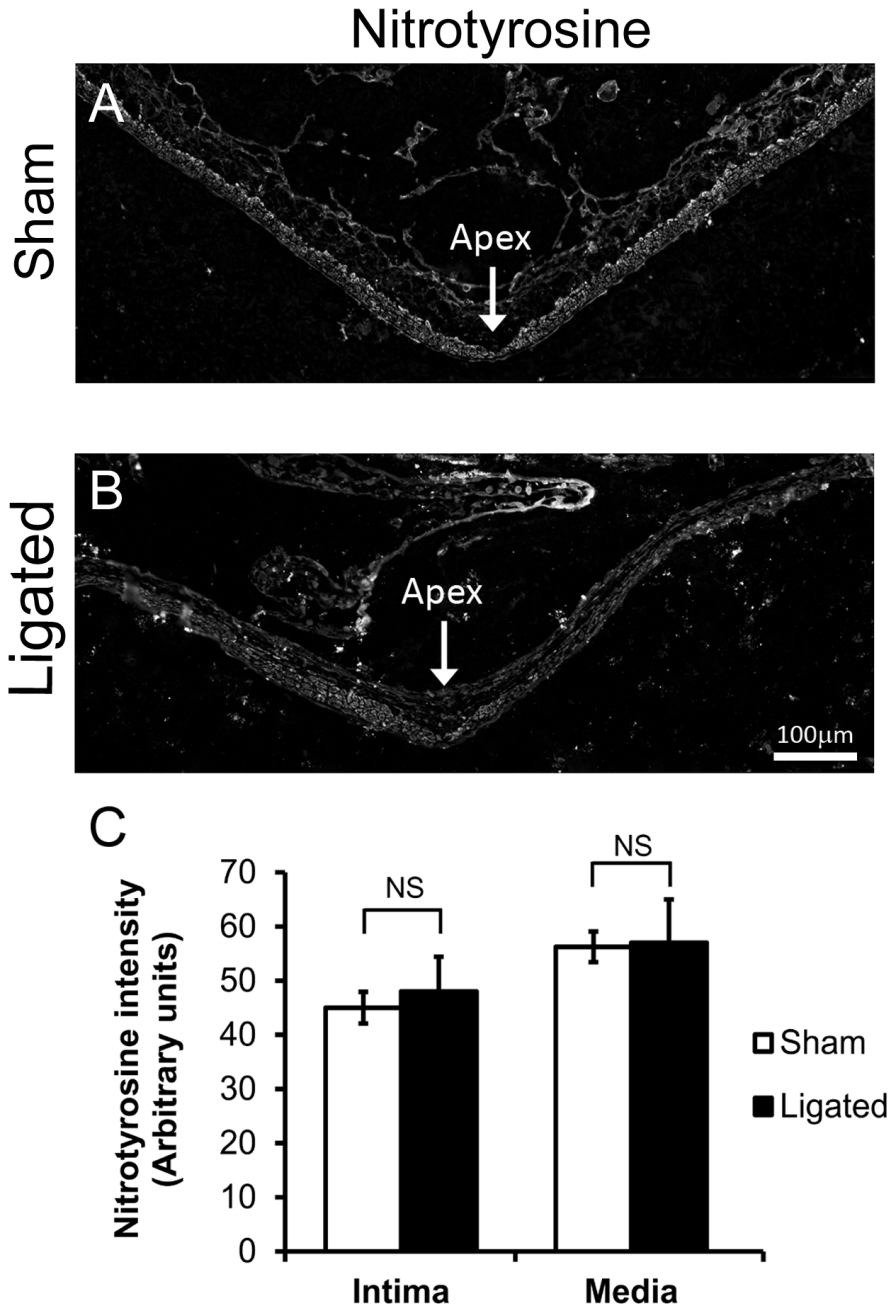
Lack of peroxynitrite formation during flow-induced aneurysm initiation. The BTs were harvested from rabbits 5 days after receiving either sham surgery or bilateral carotid ligation, then stained for the peroxynitrite marker, nitrotyrosine. (A) Immunofluorescent staining of nitrotyrosine at the apex of sham-operated animals compared to (B) nitrotyrosine in an area of aneurysmal damage at the BT. Scale bar = 100 µm. (C) Average intensity of immunostaining of nitrotyrosine did not change in the initima or the media of the BT. Bars represent mean ± standard error. NS indicates no statistically significant difference between ligated and sham surgery groups (p≤0.05, Mann-Whitney U-test).

NOS can cause cell injury by producing peroxynitrite, a protein- and DNA- damaging anion formed when NO reacts with superoxide [23]. Therefore, we tested for changes in peroxynitrite by staining for one of its reaction products, nitrotyrosine (Figure 4). Nitrotyrosine was detected throughout all layers of the BT in both sham-operated and 5-day ligated animals (Figure 4A and 4B). However, measurement of nitrotyrosine staining, specifically in the intimal and medial layers, indicated no significant difference in intensity between the two groups (Figure 4C; intima, p = 1 and media, p = 0.902) suggesting that NOS-mediated damage is not from peroxynitrite.

### IA-initiating hemodynamics increased ROS, but inhibition with LNAME did not affect superoxide production

**Figure 5:**
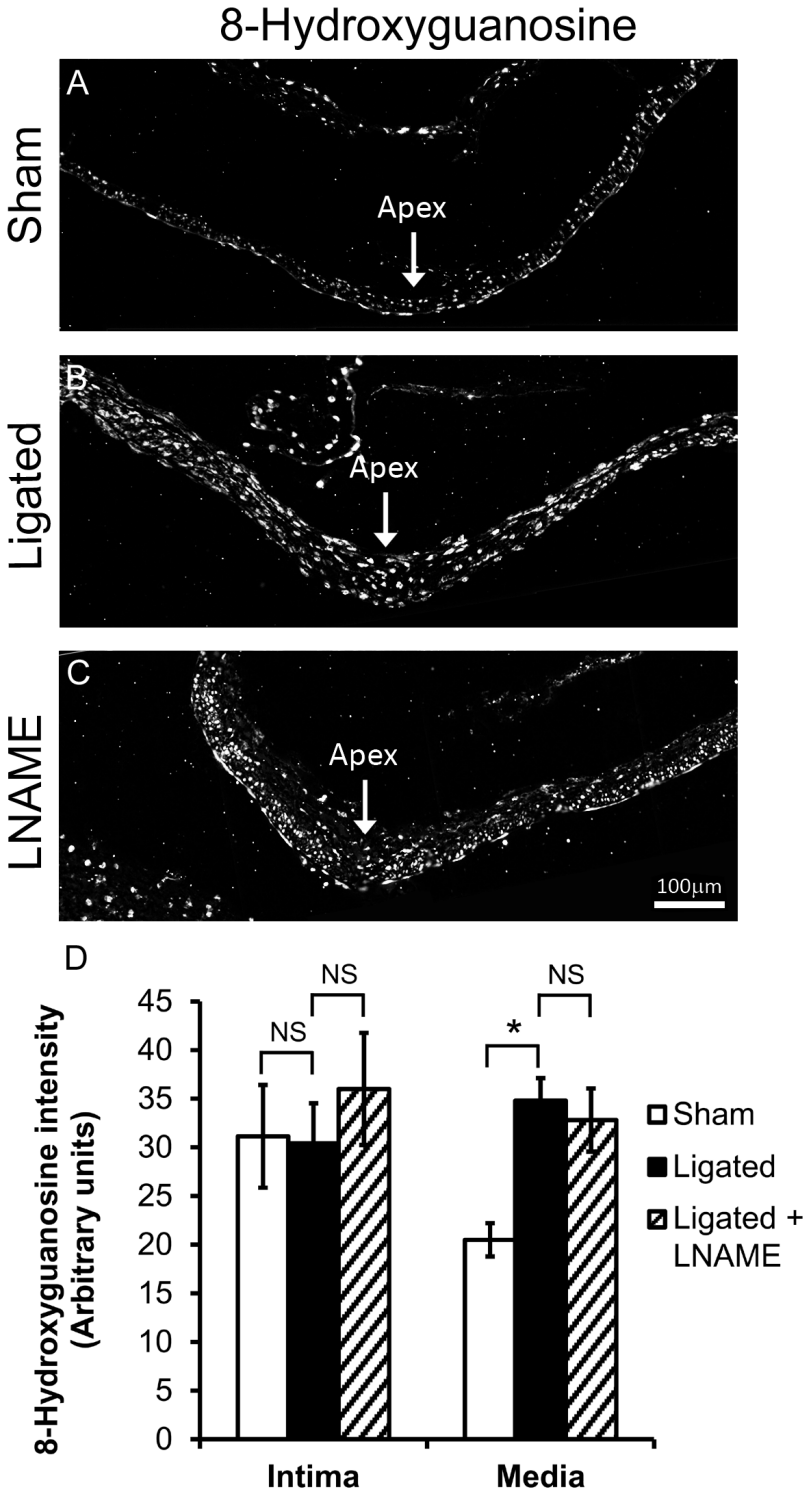
Superoxide increased in the media of the BT 5 days after ligation. 8-hydroxyguanosine, a marker of ROS, was detected by indirect immunofluorescence at the BT of rabbits that received: (A) sham surgery, (B) bilateral CCA ligation, and (C) ligation with LNAME treatment. Scale bar = 100 µm. D) 8-hydroxyguanosine was significantly higher in the media, but not in the intima of ligated animals compared to sham-operated animals. Compared to ligated rabbits, LNAME treatment did not change 8-hydroxyguanosine levels in the intima or the media of the BT. Bars represent mean ± standard error. *Statistically significant difference between groups (p≤0.05, Mann-Whitney U-test). NS indicates no significant difference.

Given that peroxynitrite was not elevated, we next asked if eNOS caused aneurysm damage because it was uncoupled and producing superoxide instead of NO [24]. BT tissue was stained for 8-hydroxyguanosine, a product of DNA oxidation by superoxide (Figure 5). 8-hydroxyguanosine was present in all layers of the BT in ligated animals vs. sham-operated animals (Figure 5B and 5A). Quantitation of the average fluorescence intensity in regions of aneurysmal damage (Figure 5D) showed a significant increase of 8-hydroxyguanosine in the media (p = 0.012) but no significant change in the intima (p = 1). However, when we stained for 8-hydroxyguanosine in NOS-inhibited animals (Figure 5C), there was no difference in staining between animals treated with LNAME and untreated ligated animals (Figure 5C vs. 5B). Quantitative analysis further confirmed that 8-hydroxyguanosine (Figure 5D) did not significantly change in either the intima (p = 0.531) or the media (p = 0.753). This suggests that (a) the elevated superoxide was not produced by eNOS, and (b) the eNOS-dependent IA damage was not mediated by superoxide.

### Scavenging superoxide decreased IA initiation

**Figure 6:**
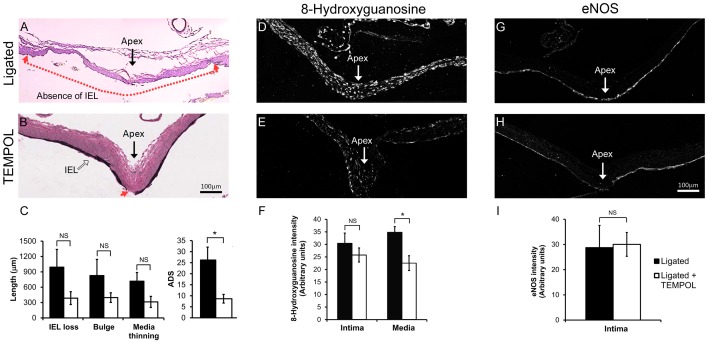
TEMPOL inhibition of flow-induced aneurysm initiation at the BT. A) Van Gieson’s stain of the BT 5 days after bilateral CCA ligation shows extensive IEL loss, slight bulge formation, and medial thinning (between red arrows). B) BT in TEMPOL-treated animals 5 days after ligation showed only slight IEL loss (red arrow) and minimal bulge formation and medial thinning. (C) The composite ADS was significantly lower 5 days after ligation in TEMPOL-treated versus untreated ligated animals. The lengths of IEL loss, bulging, and media thinning were all reduced, but these differences did not reach statistical significance. (D) Immunofluorescent staining of 8-hydroxyguanosine at the BT of CCA ligated animals compared to (E) ligated animals treated with TEMPOL. (F) Average intensity of 8-hydroxyguanosine was significantly lower in the media of TEMPOL-treated animals. (G) Immunofluorescent staining of eNOS at the BT of CCA ligated animals compared to (H) eNOS in ligated animals treated with TEMPOL. (I) There was no difference in eNOS intimal staining between animals with versus without TEMPOL. Scale bar = 100 µm. Bars in graphs represent mean ± standard error. * indicates statistically significant difference between groups (p≤0.05, Mann-Whitney U-test); NS indicates no significant difference.

To determine if the elevated superoxide observed in the media contributed to IA initiation, ligated animals were treated with TEMPOL (Figure 6), which depletes superoxide by dismutation and/or scavenging. Depletion of superoxide was confirmed by examining 8-hydroxyguanosine in the BT of TEMPOL-treated and untreated ligated animals (Figure 6D vs. 6E). Immunofluorescent staining (Figure 6F) showed significantly lower levels of 8-hydroxyguanosine in the media (p = 0.048) in TEMPOL-treated animals. 8-hydroxyguanosine staining was also reduced in the intima by TEMPOL, but this difference was not statistically significant (p = 0.539). This depletion of superoxide was accompanied by a decrease in the severity of aneurysm formation at the BT (Figure 6A vs. 6B). All of the indicators of aneurysmal damage – IEL loss, bulge, and media thinning – were lower in TEMPOL-treated animals. Although the individual decreases were not statistically significant (p = 0.143, p = 0.329, and p = 0.104, respectively), the composite ADS was significantly decreased by TEMPOL (Figure 6C; p = 0.034). Furthermore, although there was some local patchiness in eNOS distribution, there was no difference in overall eNOS staining intensity between ligated animals treated with TEMPOL and untreated ligated animals (Figure 6G vs. 6H). Quantitative analysis (Figure 6I) confirmed that eNOS staining did not significantly change in the intima across the portion of the BT where hemodynamic aneurysm initiation normally occurs after carotid ligation (p = 0.691).

### TEMPOL and LNAME treatments decreased SMC de-differentiation and MMP protein levels

**Figure 7:**
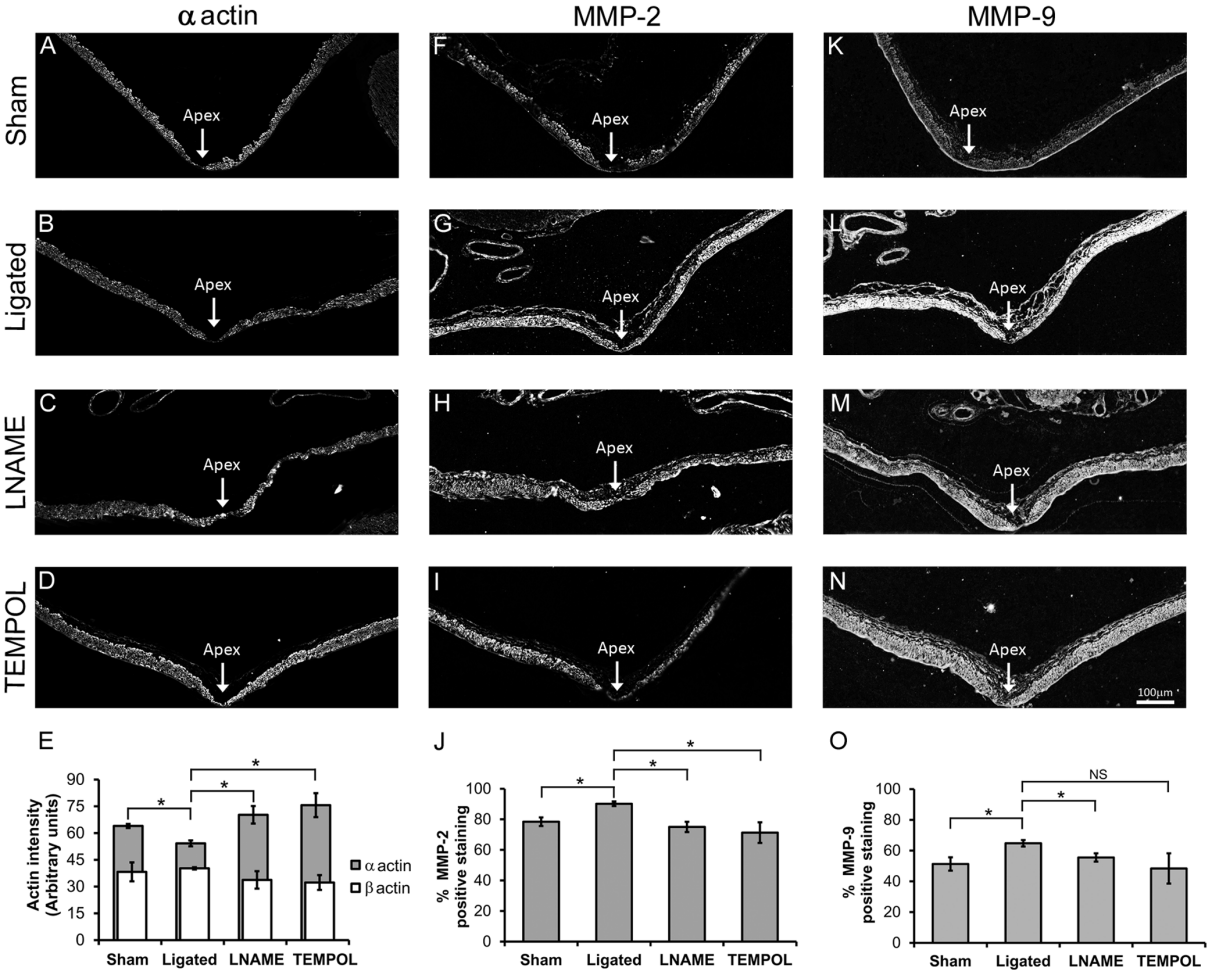
LNAME and TEMPOL restored α-actin and decreased MMPs at the BT of ligated animals. Smooth muscle α-actin, MMP-2 and MMP-9 proteins were detected by immunofluorescence in sham-operated animals (A, F, K), and in CCA ligated animals given no inhibitors (B, G, L), or treated with LNAME (C, H, M), or TEMPOL (D, I, N). α-actin staining in the media significantly decreased at the BT following ligation (B) compared to sham-operated animals (A), but was restored with either LNAME (C) or TEMPOL (D) treatment. MMP-2 staining increased at the BT following ligation (G) compared to the sham surgery group (F), and was decreased with either LNAME (H) or TEMPOL (I) treatment. Similarly, MMP-9 staining in the media increased at the BT following ligation (L) compared to sham-operated animals (K), and was decreased with either LNAME (M) or TEMPOL (N) treatment. Scale bar = 100 µm. Panels E, J and O show quantitative analysis of these stainings. (E) Intensity of α-actin and β-actin were measured in SMCs at the BT. α-actin was decreased by ligation but increased with both LNAME and TEMPOL treatments while β-actin did not change among groups. Quantification of MMP-2 (J) and MMP-9 (O) immunofluorescence indicated significant changes in the percent area of positive staining at the BT. While TEMPOL treatment decreased MMP-9 staining compared to untreated ligated animals the change was not statistically significant. Bars represent mean ± standard error. *Statistically significant difference between groups (p≤0.05, Mann-Whitney U-test). NS indicates no significant difference.

To further understand the roles of eNOS and superoxide in aneurysm initiation, we next tested if eNOS activity and superoxide affected MMP production and SMC phenotype. For this purpose, we examined α-actin, a marker of contractile phenotype, in SMCs at the BT of sham-operated, ligated and drug treated animal groups. Consistent with previous results [12], the BT of ligated animals had weaker α-actin staining as compared to sham-operated animals (Figure 7A, B, E; p = 0.028). This indicates deviation from the usual SMC contractile phenotype. α-actin staining in ligated animals treated with LNAME was similar to sham-operated animals (Figure 7A vs. 7C), and α-actin was significantly higher in LNAME-treated vs. ligated animals with no drug treatment (Figure 7E; p = 0.041). In a similar manner, ligated animals treated with TEMPOL showed significantly more α-actin (Figure 7D) when compared to untreated ligated animals (Figure 7D vs 7B and 7E; p = 0.03). To confirm that α-actin levels did not change because of SMC loss, we stained for β-actin and found that β-actin remains relatively constant among groups (Figure 7E).

As previously demonstrated [10], [12], MMP-2 and MMP-9 protein were present and high in regions adjacent to the apex of the BT in ligated animals (Figure 7G, K to 7L). The proportion of the vessel wall that stained positively for MMP-2 and MMP-9 was significantly higher in ligated animals than those receiving sham surgery (Figure 7J; p = 0.020 and Figure 7O; p = 0.037, respectively). LNAME inhibited this response for MMP-2 and MMP-9 (compare Figure 7G to 7H and Figure 7L to 7M, respectively). The percent area of tissue staining positively for MMP-2 and MMP-9 was significantly reduced in LNAME treated animals (Figure 7J; p = 0.030 and Figure 7O; p = 0.037, respectively). Treatment with TEMPOL also decreased both MMP-2 and MMP-9 (Figure 7I and 7N, respectively). This reduction was significant for MMP-2 (Figure 7J; p = 0.037), but not for MMP-9 (Figure 7O; p = 0.296).

## Discussion

The results of the current study strongly suggest that under hemodynamic insult, increases in eNOS activity and/or superoxide production contribute to IA initiation. Both molecules affect SMC phenotype, increasing MMP production and aneurysmal damage.

### eNOS activity and superoxide production are both effectors of hemodynamic IA initiation

Our results indicate that NOS activity reduces smooth muscle α-actin and increases MMPs during flow-induced IA formation. Specifically, eNOS, and not iNOS or nNOS, was significantly upregulated during aneurysm initiation, highlighting eNOS as the mediator of IA initiation. The relative importance of eNOS over the other NOS isoforms is consistent with the hemodynamic and non-inflammatory nature of the IA initiation model. nNOS is constitutively expressed in vascular cells but does not respond to increased wall shear stress [25]. iNOS expression and activity is associated with macrophage infiltration into the vessel wall, which is typically seen in well-developed human aneurysms or weeks to months after aneurysm induction in animals. Previous studies in this rabbit IA model show little or no inflammatory cell infiltration during early aneurysm remodeling, and when inflammatory cells were present, they were found exclusively in the adventitia and not localized to the area of IA initiation [10], [12]. Furthermore, systemic depletion of macrophages fails to inhibit IA initiation [12]. The high wall shear stress environment under which aneurysms first initiate is not conducive for inflammatory cell infiltration, which requires substantial blood residence time. In contrast, eNOS expression and activity are flow sensitive and have been shown to increase under chronically elevated wall shear stress [21], [26]. Thus, eNOS is well-positioned to act as an effecter of hemodynamic aneurysm initiation.

Although eNOS primarily generates NO, when uncoupled it can produce superoxide. Superoxide generated from eNOS uncoupling has been associated with vascular diseases such as atherosclerosis, hypertension and diabetes [27]. In the current study however, it appears that flow-induced superoxide was not generated by eNOS. ROS did not change in LNAME treated animals. Because of its structural similarity to L-arginine, LNAME can bind NOS to competitively inhibit NO production in the coupled state and superoxide production in the uncoupled state. Furthermore, in our experiments, ROS was only elevated in the media, whereas eNOS increased exclusively in the intima. From these results we deduce that during IA initiation, eNOS does not produce ROS and that LNAME inhibits aneurysm damage by inhibiting the production of NO.

Although eNOS was not responsible for the elevated superoxide observed during IA initiation, superoxide did contribute to IA initiation. Superoxide scavenging via TEMPOL significantly reduced ROS production in the media and attenuated aneurysm development. ROS is believed to affect SMC phenotypic, causing a change from a contractile phenotype to “synthetic” phenotype. When synthetic, SMCs produce matrix components, upregulate MMPs and inflammatory cytokines, and exhibit increased proliferation and migration – responses associated with atherosclerotic lesion development [28], [29]. Our data indicate that superoxide may also modulate SMC phenotype during IA development. SMC de-differentiation in response to flow-induced superoxide may facilitate IEL damage and medial thinning through MMP upregulation. It is interesting to note that NADPH oxidase has been found upregulated in the vessel wall, including the media, of hypertensive rodents during IA formation [30], and this superoxide generator has been associated with decreased SMC contractile markers [31]. Thus, NADPH oxidase may be of particular importance in follow-up studies to further elucidate the role of superoxide in flow-mediated aneurysm initiation.

Because NO and superoxide both contribute to IA initiation by increasing MMPs both molecules could be acting through some common mediator. In other systems, such a role has been ascribed to peroxynitrite, a cytotoxic agent that elicits a range of cellular responses, from minor changes in cell signaling to necrosis and apoptosis [23]. However, at the BT, peroxynitrite levels did not change under aneurysm-inducing flow conditions. An alternative candidate is NF-κB, a transcriptional regulator of MMPs that is found in SMCs of hemodynamically induced IAs [12] and which is activated by both NO [32] and superoxide [33]. Further studies are required to determine whether NO and superoxide affect SMCs via a common mediator or through independent signaling mechanisms.

### Role of eNOS-derived NO in flow-induced IA initiation

eNOS-derived NO is conventionally considered a protective molecule in the vasculature – it facilitates vasodilation and prevents platelet aggregation and leukocyte attachment to the endothelium [34]. Surprisingly, however, we found that eNOS activity causes vascular damage, promoting increased MMPs and facilitating IA initiation. Moreover, inhibiting eNOS did not affect superoxide levels, so the responsible eNOS product was presumably NO.

The damaging effect of NO in our hemodynamics-only IA model may be analogous to responses seen in flow-loaded arteries that undergo non-aneurysmal, adaptive expansive remodeling. Outward expansion of a flow-loaded straight vessel is facilitated by endothelium-derived NO activity. Specifically, NO promotes the upregulation of MMP-2 and MMP-9 in ECs and SMCs to allow for arterial distensibility by fragmenting the IEL [14], [35]. Furthermore, during expansive remodeling, SMCs display a non-contractile phenotype [36]–[38], and inflammatory cells are scant [37], [39], much as we observe in early stages of an IA. Thus, under high flow conditions, NO can promote vascular remodeling through mural cell-mediated mechanisms.

In contrast to our study, a protective effect of eNOS during IA development has been found in rodents. Specifically, mice with double knockout of eNOS and nNOS [13] have increased IA formation compared to wild type animals. However, rodents were primed with additional risk factors, including high salt diet and renal artery ligation to induce hypertension, which can upregulate inflammatory cytokines and promote inflammatory cell infiltration [40]. The eNOS-knockout mice are likely to be more susceptible to aneurysm initiation because of the pro-inflammatory effects of hypertension. Indeed, inflammatory cell infiltration and macrophage-derived MMPs have been found to facilitate aneurysm formation in that model [41], [42].

The role of NO in IA initiation is thus complex. NO alters cell adhesion mechanisms in endothelial cells to prevent leukocyte attachment [34]. In conditions that promote vascular inflammation (e.g., hypertension) eNOS levels are reduced in the endothelium [13], [43], vessels become prone to infiltration, and inflammatory cells are found in the wall at aneurysm initiation sites [41], [42]. The complete loss of NO in eNOS knockout mice would exacerbate this effect, and so the greater aneurysmal damage observed in such models may be due to inflammatory infiltration rather than SMC activity [13]. In contrast, under flow-loading conditions in normal animals, eNOS levels are increased [21], [26], which may explain the lack of early inflammatory infiltration in aneurysms induced by flow alone [10], [12]. Meanwhile, endothelial produced NO diffusing to neighboring SMCs can induce phenotype change and MMP production. It is then SMC-derived MMPs that drive aneurysmal damage. Thus, there may be different NO-mediated routes to aneurysm development, depending on the initial health of the vessel.

### NO and superoxide may have additive or synergistic effects on aneurysmal damage

It is clear that both eNOS activity and superoxide are effectors of IA initiation. It is interesting to note that when eNOS activity was inhibited, ROS was still elevated; yet IA damage was substantially reduced. Likewise, IA formation was inhibited when superoxide was depleted without any treatment to block NOS. Thus it appears that neither signaling molecule elicits a full aneurysmal response on its own. IA initiation may be likened to a marble spilling from a bowl [44], [45]. A vessel homeostasis exists when the marble sits in the bottom of the bowl, and aneurysm-initiating factors are perturbations of the marble. If the perturbation is insufficient to move the marble over the edge of the bowl, the marble is able to return to equilibrium, but when the marble is pushed over the edge, irreversible progression to aneurysm occurs. Pre-existing IA risk factors such as hypertension, smoking, and genetic predisposition, would decrease the height of the bowl edge, so that smaller perturbations can result in aneurysm. NOS activity and superoxide represent forces that can perturb the marble, and together, push the marble over the edge. In our system when one is absent (inhibited), the force is no longer sufficient to push the marble over the edge, and IA is less likely to occur.

## Conclusion

NO production from eNOS and superoxide can both contribute to mural damage leading to IA initiation. Both molecules are involved in eliciting SMC phenotype change and MMP production in response to hemodynamic insult to promote aneurysm damage.

## References

[1] Fujiwara S, Fujii K, Fukui M (1993) De novo aneurysm formation and aneurysm growth following therapeutic carotid occlusion for intracranial internal carotid artery (ICA) aneurysms. Acta Neurochir (Wien) 120: 20–25. 8434512 10.1007/BF02001464

[2] Hashimoto T, Meng H, Young WL (2006) Intracranial aneurysms: links among inflammation, hemodynamics and vascular remodeling. Neurol Res 28: 372–380. 16759441 10.1179/016164106X14973 PMC2754184

[3] Stehbens WE (1989) Etiology of intracranial berry aneurysms. J Neurosurg 70: 823–831. 2654334 10.3171/jns.1989.70.6.0823

[4] Wolf RL, Imbesi SG, Galetta SL, Hurst RW, Sinson GP, et al (2002) Development of a posterior cerebral artery aneurysm subsequent to occlusion of the contralateral internal carotid artery for giant cavernous aneurysm. Neuroradiology 44: 443–446. 12012132 10.1007/s00234-001-0723-5

[5] Briganti F, Cirillo S, Caranci F, Esposito F, Maiuri F (2002) Development of “de novo” aneurysms following endovascular procedures. Neuroradiology 44: 604–609. 12136363 10.1007/s00234-001-0732-4

[6] Suzuki MT, Aguiar GB, Jory M, Conti ML, Veiga JC (2011) De novo basilar tip aneurysm. Case report and literature review. Neurocirugia (Astur) 22: 251–254. 21743946 10.1016/s1130-1473(11)70020-3

[7] Timperman PE, Tomsick TA, Tew JM, van Loveren HR (1995) Aneurysm formation after carotid occlusion. American Journal of Neuroradiology 16: 329–331. 7726081 PMC8338346

[8] Gao L, Hoi Y, Swartz DD, Kolega J, Siddiqui A, et al (2008) Nascent aneurysm formation at the basilar terminus induced by hemodynamics. Stroke 39: 2085–2090. 18451348 10.1161/STROKEAHA.107.509422 PMC2559803

[9] Meng H, Metaxa E, Gao L, Liaw N, Natarajan SK, et al (2011) Progressive aneurysm development following hemodynamic insult. J Neurosurg 114: 1095–1103. 20950086 10.3171/2010.9.JNS10368

[10] Kolega J, Gao L, Mandelbaum M, Mocco J, Siddiqui AH, et al (2011) Cellular and molecular responses of the basilar terminus to hemodynamics during intracranial aneurysm initiation in a rabbit model. J Vasc Res 48: 429–442. 21625176 10.1159/000324840 PMC3121554

[11] Metaxa E, Tremmel M, Natarajan SK, Xiang J, Paluch RA, et al (2010) Characterization of critical hemodynamics contributing to aneurysmal remodeling at the basilar terminus in a rabbit model. Stroke 41: 1774–1782. 20595660 10.1161/STROKEAHA.110.585992 PMC2913882

[12] Mandelbaum M, Kolega J, Dolan JM, Siddiqui AH, Meng H (2013) A critical role for proinflammatory behavior of smooth muscle cells in hemodynamic initiation of intracranial aneurysm. PLoS One 8: e74357. 24023941 10.1371/journal.pone.0074357 PMC3759467

[13] Aoki T, Nishimura M, Kataoka H, Ishibashi R, Nozaki K, et al (2011) Complementary inhibition of cerebral aneurysm formation by eNOS and nNOS. Lab Invest 91: 619–626. 21321533 10.1038/labinvest.2010.204

[14] Tronc F, Mallat Z, Lehoux S, Wassef M, Esposito B, et al (2000) Role of matrix metalloproteinases in blood flow-induced arterial enlargement: interaction with NO. Arterioscler Thromb Vasc Biol. 20: E120–126. 10.1161/01.atv.20.12.e120 11116076

[15] Gurjar MV, DeLeon J, Sharma RV, Bhalla RC (2001) Mechanism of inhibition of matrix metalloproteinase-9 induction by NO in vascular smooth muscle cells. J Appl Physiol 91: 1380–1386. 11509539 10.1152/jappl.2001.91.3.1380

[16] Nelson KK, Melendez JA (2004) Mitochondrial redox control of matrix metalloproteinases. Free Radic Biol Med 37: 768–784. 15304253 10.1016/j.freeradbiomed.2004.06.008

[17] Lee MY, Griendling KK (2008) Redox signaling, vascular function, and hypertension. Antioxid Redox Signal 10: 1045–1059. 18321201 10.1089/ars.2007.1986 PMC2828811

[18] Sung HJ, Eskin SG, Sakurai Y, Yee A, Kataoka N, et al (2005) Oxidative stress produced with cell migration increases synthetic phenotype of vascular smooth muscle cells. Ann Biomed Eng 33: 1546–1554. 16341922 10.1007/s10439-005-7545-2

[19] Hoi Y, Gao L, Tremmel M, Paluch RA, Siddiqui AH, et al (2008) In vivo assessment of rapid cerebrovascular morphological adaptation following acute blood flow increase. Journal of Neurosurgery 109: 1141–1147. 19035734 10.3171/JNS.2008.109.12.1141 PMC2775477

[20] Schiffrin EL (1992) Reactivity of small blood vessels in hypertension: relation with structural changes. State of the art lecture. Hypertension 19: Ii1–9. 10.1161/01.hyp.19.2_suppl.ii1-a 1735561

[21] Dumont O, Loufrani L, Henrion D (2007) Key role of the NO-pathway and matrix metalloprotease-9 in high blood flow-induced remodeling of rat resistance arteries. Arterioscler Thromb Vasc Biol 27: 317–324. 17158349 10.1161/01.ATV.0000254684.80662.44 PMC2234579

[22] Guzman RJ, Abe K, Zarins CK (1997) Flow-induced arterial enlargement is inhibited by suppression of nitric oxide synthase activity in vivo. Surgery 122: 273–279 discussion 279–280. 9288132 10.1016/s0039-6060(97)90018-0

[23] Pacher P, Beckman JS, Liaudet L (2007) Nitric oxide and peroxynitrite in health and disease. Physiol Rev 87: 315–424. 17237348 10.1152/physrev.00029.2006 PMC2248324

[24] Vasquez-Vivar J, Kalyanaraman B, Martasek P, Hogg N, Masters BS, et al (1998) Superoxide generation by endothelial nitric oxide synthase: the influence of cofactors. Proc Natl Acad Sci U S A 95: 9220–9225. 9689061 10.1073/pnas.95.16.9220 PMC21319

[25] Melikian N, Seddon MD, Casadei B, Chowienczyk PJ, Shah AM (2009) Neuronal nitric oxide synthase and human vascular regulation. Trends Cardiovasc Med 19: 256–262. 20447567 10.1016/j.tcm.2010.02.007 PMC2984617

[26] Metaxa E, Meng H, Kaluvala SR, Szymanski MP, Paluch RA, et al (2008) Nitric oxide-dependent stimulation of endothelial cell proliferation by sustained high flow. Am J Physiol Heart Circ Physiol 295: H736–742. 18552158 10.1152/ajpheart.01156.2007 PMC2519227

[27] Fukai T, Folz RJ, Landmesser U, Harrison DG (2002) Extracellular superoxide dismutase and cardiovascular disease. Cardiovasc Res 55: 239–249. 12123763 10.1016/s0008-6363(02)00328-0

[28] Gomez D, Owens GK (2012) Smooth muscle cell phenotypic switching in atherosclerosis. Cardiovasc Res 95: 156–164. 22406749 10.1093/cvr/cvs115 PMC3388816

[29] Orr AW, Hastings NE, Blackman BR, Wamhoff BR (2010) Complex regulation and function of the inflammatory smooth muscle cell phenotype in atherosclerosis. J Vasc Res 47: 168–180. 19851078 10.1159/000250095 PMC2842170

[30] Aoki T, Nishimura M, Kataoka H, Ishibashi R, Nozaki K, et al (2009) Reactive oxygen species modulate growth of cerebral aneurysms: a study using the free radical scavenger edaravone and p47phox(−/−) mice. Lab Invest 89: 730–741. 19381132 10.1038/labinvest.2009.36

[31] West N, Guzik T, Black E, Channon K (2001) Enhanced superoxide production in experimental venous bypass graft intimal hyperplasia: role of NAD(P)H oxidase. Arterioscler Thromb Vasc Biol 21: 189–194. 11156851 10.1161/01.atv.21.2.189

[32] McAdam E, Haboubi HN, Forrester G, Eltahir Z, Spencer-Harty S, et al (2012) Inducible nitric oxide synthase (iNOS) and nitric oxide (NO) are important mediators of reflux-induced cell signalling in esophageal cells. Carcinogenesis 33: 2035–2043. 22826608 10.1093/carcin/bgs241

[33] Wang S, Leonard SS, Castranova V, Vallyathan V, Shi X (1999) The role of superoxide radical in TNF-alpha induced NF-kappaB activation. Ann Clin Lab Sci 29: 192–199. 10440583

[34] Forstermann U, Munzel T (2006) Endothelial nitric oxide synthase in vascular disease: from marvel to menace. Circulation 113: 1708–1714. 16585403 10.1161/CIRCULATIONAHA.105.602532

[35] Castier Y, Brandes RP, Leseche G, Tedgui A, Lehoux S (2005) p47phox-dependent NADPH oxidase regulates flow-induced vascular remodeling. Circ Res 97: 533–540. 16109921 10.1161/01.RES.0000181759.63239.21

[36] Buus CL, Pourageaud F, Fazzi GE, Janssen G, Mulvany MJ, et al (2001) Smooth muscle cell changes during flow-related remodeling of rat mesenteric resistance arteries. Circ Res 89: 180–186. 11463726 10.1161/hh1401.093575

[37] Jones GT, Stehbens WE, Martin BJ (1994) Ultrastructural changes in arteries proximal to short-term experimental carotid-jugular arteriovenous fistulae in rabbits. Int J Exp Pathol 75: 225–232. 8086318 PMC2001805

[38] Sho E, Komatsu M, Sho M, Nanjo H, Singh TM, et al (2003) High flow drives vascular endothelial cell proliferation during flow-induced arterial remodeling associated with the expression of vascular endothelial growth factor. Exp Mol Pathol 75: 1–11. 12834620 10.1016/s0014-4800(03)00032-7

[39] Nuki Y, Matsumoto MM, Tsang E, Young WL, van Rooijen N, et al (2009) Roles of macrophages in flow-induced outward vascular remodeling. J Cereb Blood Flow Metab 29: 495–503. 19002198 10.1038/jcbfm.2008.136 PMC2649678

[40] Montezano AC, Touyz RM (2012) Molecular mechanisms of hypertension–reactive oxygen species and antioxidants: a basic science update for the clinician. Can J Cardiol 28: 288–295. 22445098 10.1016/j.cjca.2012.01.017

[41] Aoki T, Kataoka H, Ishibashi R, Nozaki K, Egashira K, et al (2009) Impact of monocyte chemoattractant protein-1 deficiency on cerebral aneurysm formation. Stroke 40: 942–951. 19164781 10.1161/STROKEAHA.108.532556

[42] Aoki T, Kataoka H, Morimoto M, Nozaki K, Hashimoto N (2007) Macrophage-derived matrix metalloproteinase-2 and -9 promote the progression of cerebral aneurysms in rats. Stroke 38: 162–169. 17122420 10.1161/01.STR.0000252129.18605.c8

[43] Fukuda S, Hashimoto N, Naritomi H, Nagata I, Nozaki K, et al (2000) Prevention of rat cerebral aneurysm formation by inhibition of nitric oxide synthase. Circulation 101: 2532–2538. 10831529 10.1161/01.cir.101.21.2532

[44] Dolan JM, Kolega J, Meng H (2013) High wall shear stress and spatial gradients in vascular pathology: a review. Ann Biomed Eng 41: 1411–1427. 23229281 10.1007/s10439-012-0695-0 PMC3638073

[45] Meng H, Xiang J, Liaw N (2012) The role of hemodynamics in intracranial aneurysm initiation. International Review of Thrombosis 7: 40–57.

